# β-Diversity of Functional Groups of Woody Plants in a Tropical Dry Forest in Yucatan

**DOI:** 10.1371/journal.pone.0073660

**Published:** 2013-09-10

**Authors:** Jorge Omar López-Martínez, Lucía Sanaphre-Villanueva, Juan Manuel Dupuy, José Luis Hernández-Stefanoni, Jorge Arturo Meave, José Alberto Gallardo-Cruz

**Affiliations:** 1 Centro de Investigación Científica de Yucatán, Mérida, México; 2 Departamento de Ecología y Recursos Naturales, Facultad de Ciencias, Universidad Nacional Autónoma de México, México, México; Centre National de la Recherche Scientifique, France

## Abstract

Two main theories have attempted to explain variation in plant species composition (β-diversity). Niche theory proposes that most of the variation is related to environment (environmental filtering), whereas neutral theory posits that dispersal limitation is the main driver of β-diversity. In this study, we first explored how α- and β-diversity of plant functional groups defined by growth form (trees, shrubs and lianas, which represent different strategies of resource partitioning), and dispersal syndrome (autochory, anemochory and zoochory, which represent differences in dispersal limitation) vary with successional age and topographic position in a tropical dry forest. Second, we examined the effects of environmental, spatial, and spatially-structured environmental factors on β-diversity of functional groups; we used the spatial structure of sampling sites as a proxy for dispersal limitation, and elevation, soil properties and forest stand age as indicators of environmental filtering. We recorded 200 species and 22,245 individuals in 276 plots; 120 species were trees, 41 shrubs and 39 lianas. We found that β-diversity was highest for shrubs, intermediate for lianas and lowest for trees, and was slightly higher for zoochorous than for autochorous and anemochorous species. All three dispersal syndromes, trees and shrubs varied in composition among vegetation classes (successional age and topographic position), whilst lianas did not. β-diversity was influenced mostly by proxies of environmental filtering, except for shrubs, for which the influence of dispersal limitation was more important. Stand age and topography significantly influenced α-diversity across functional groups, but showed a low influence on β-diversity –possibly due to the counterbalancing effect of resprouting on plant distribution and composition. Our results show that considering different plant functional groups reveals important differences in both α- and β-diversity patterns and correlates that are not apparent when focusing on overall woody plant diversity, and that have important implications for ecological theory and biodiversity conservation.

## Introduction

Uncovering the mechanisms underlying variation in species composition (β-diversity) is a central goal of community ecology and biogeography [1], [2], [3]. Different theories have been proposed to explain variation in species composition, and many of our current ideas are based on the observation that species differ in their environmental requirements [4], [5]. The niche theory proposes that most of this variation is related to particular adaptations of species to the environment [6], [7], [8]. Therefore, species abundance may be an indicator of how suitable environmental conditions are for species to grow and reproduce. In this sense, functional attributes such as growth form would be important to understand the mechanisms assembling plant communities, because they represent different strategies of resource partitioning [4].

In contrast, the neutral theory [9], [10], [11] suggests that all species are competitively equal and able to grow equally well under a wide range of environmental conditions. According to the neutral theory, the main factor determining plant community composition is dispersal limitation or recruitment limitation, that is, the limited capacity of plants to effectively disperse their seeds and establish seedlings away from parent individuals, which causes the spatial location of an individual to be constrained by the location of its parent. As a result, sites located closer together should be more similar in composition than those lying far apart [10], [12] regardless of their environmental conditions [13]. Therefore, dispersal syndrome appears to be an important functional trait that should be considered to understand the mechanisms affecting β-diversity.

Other factors may also influence β-diversity patterns. For example, previous studies have shown that the magnitude of β-diversity is influenced by secondary succession [14], soil properties [1], [2], [15], [16], fragmentation [17] and topography [18]. These factors produce changes in community patterns by modifying the local environment and resource availability [19], hence creating opportunities for the establishment of new species and reducing populations of those that are already established (see reviews by [20], [21], [22]).

Additionally, species with specific traits may be favored over others at different successional stages, and different ecological strategies are dominant at different moments of vegetation development [23], [24], [25]. For instance, it has been found that plants of early successional stages in Neotropical lowland forests have primarily anemochorous (i.e. wind-dispersed) or autochorous (explosive) dispersal, while plants with zoochorous (i.e. animal-dispersed) seeds are more common in old-growth forests [26], [27]. Similarly, different life forms alternate dominance along secondary succession. In tropical wet forests, herbs, shrubs and vines dominate during the initial successional stages, and are subsequently replaced by short- and long-lived pioneer trees – which in turn are replaced by shade-tolerant trees [28]. In Bolivian dry forests the abundance of shrubs, lianas and bromeliads increases with successional age, whereas grasses become increasingly rarer [29]. Moreover, it has been proposed that species with long-distance dispersal, such as pioneer species, should be able to locate most of the appropriate habitats in a landscape, and such dispersal may compensate for low persistence rates at any given site [30], [31]. Accordingly, spatial variation in species composition (i.e. β-diversity) is expected to be lowest for species with the longest dispersal distance.

β-diversity also varies with plant community type, even within a single landscape [32]. For instance, β-diversity tended to increase when moving from a tree-dominated community to a shrub- or herb-dominated one [33]. However, β-diversity patterns among growth forms are not always consistent. For example, in Appalachian spruce-fir forests β-diversity values were twice as large for herb assemblages compared to woody plants [30], while in a Chinese subtropical forest, trees showed higher β-diversity than shrubs [34]. Thus, dispersal syndrome and growth form are important plant life-history traits that could shed light on the processes and mechanisms influencing plant community assembly [4], [35], [36].

The objectives of this study were twofold. First, we wanted to explore how α- and β-diversity of plant functional groups defined by dispersal syndrome and growth form vary with successional age and topographic position. Second, we sought to assess and compare the relative influence of environmental filtering (niche differentiation) vs. dispersal limitation on β-diversity of these functional groups. We used elevation, soil properties and forest stand age as putative environmental filters, whereas dispersal limitation was assessed in terms of geographical distance among sampling sites.

We made three predictions based on the findings of previous studies and on the following arguments. Since β-diversity is expected to be inversely related to dispersal distance [30], [31], our first prediction was that β-diversity would be highest for autochorous species and for shrubs, and lowest for anemochorus species and for trees (P1). Second, since plant diversity and structural complexity generally increase during tropical forest succession [20], [28], [37], [38], [39], we expected an increase in overall species richness (α-diversity) across growth forms and dispersal syndromes during succession, with a prevalence of shrubs and of anemochorous and autochorous species in early successional ages, and of zoochory and tree species in more advanced successional ages (P2). Finally, traits that are relevant for plants' ecological strategy could have evolved in response to environmental filters [4], [40], [41], [42]. Since a greater dispersal distance may increase the chances of finding appropriate environmental conditions for establishment, we predicted that the association between environmental filters and β-diversity would be strongest for trees and for anemochorous species, and weakest for shrubs and for autochorous species (P3).

## Materials and Methods

### Study area

**Figure 1:**
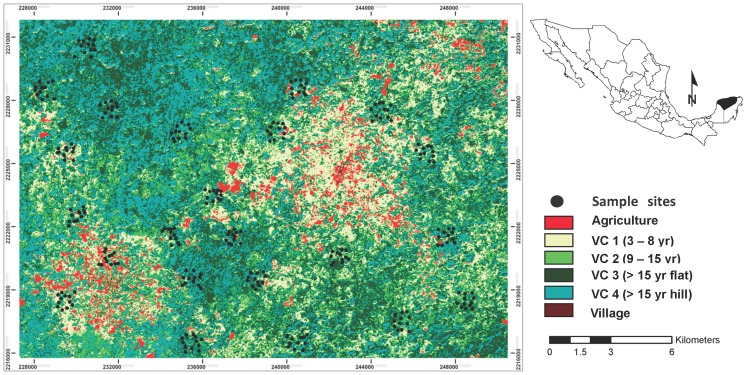
Location and land-cover thematic map of the study area showing the location of sampling sites.

The study area (ca. 22×16 km) is centrally located in the Yucatán Peninsula, Mexico (20°01′–20°16′N, 89°39′–89°60′W) (Fig. 1). Climate is warm sub-humid, with summer rain (May – October) and a marked dry season (November – April). Mean annual temperature is ca. 26°C, and mean annual precipitation ranges between 1,000 and 1,200 mm [43]. The landscape consists of Cenozoic limestone hills with moderate slopes (10–25°), which alternate with flat areas; elevation ranges from 60 to 180 m a.s.l. Soil heterogeneity is strongly associated with topography; with shallow, rocky Lithosols and Rendzines dominating the hills, while deeper, clayey Luvisols and Cambisols prevail in flat areas [44]. Regional vegetation is classified as seasonally dry tropical forest (50–75% of the species shed their leaves during the dry season) and it is composed of forest stands of different successional ages since abandonment after traditional slash-and-burn agriculture, which has been practiced for over 2000 years [45], and permanent agricultural fields concentrated around the villages (Fig. 1). Land tenure is mostly communal and approvals for field work were obtained from each of the “ejidos” whose lands fell within the study area: Xkobenhaltún, Xul and Yaxhachén.

### Vegetation and environmental data

A land cover map of the studied area was obtained from a SPOT 5 satellite image (10 m spatial resolution) acquired in January 2005, after conducting a supervised classification of previously geo-referenced and radiometrically corrected bands 2 (red), 3 (near infrared), and 4 (mid infrared). Six land-cover types were defined: (1) 3–8 yr-old secondary forest (vegetation class 1 or: VC1); (2) 9–15 yr-old secondary forest (VC2); (3) >15 yr-old secondary forest on flat areas (VC3); (4) >15 yr-old secondary forest on hills (VC4)¸ (5) agricultural fields; (6) urban areas and roads. The vegetation classes correspond to different ages of forest succession and different topographic positions. The ranges of successional ages were selected according to the differences in vegetation reflectance using the SPOT 5 imagery, while topographic position represented the hills and flat areas present in the landscape. Both attributes have been shown to influence vegetation structure, diversity and composition, with canopy height, basal area and α-diversity increasing with forest stand age, and most attributes, including species composition, differing between hills and flat areas [39]. The accuracy of the classified map was assessed using the overall accuracy and Cohen's Kappa statistic (see [46] for details on the classification procedure).

Field data were collected from June to September of 2008 and 2009 through a hierarchical design. First, we randomly selected 23 sampling landscapes (SL) of 1 km^2^ each, encompassing the whole range of forest fragmentation in the study area. We then randomly located three plots for each of the four vegetation classes (VC1-VC4) within each SL (276 sampling sites in total) using a GPS unit. Each plot consisted of a circular 200 m^2^ area where all woody plants ≥5 cm in DBH were recorded, plus a concentric 50 m^2^-subplot where all woody plants ≥1 cm in DBH were recorded. Stand age of each site was determined through interviews with local residents who had lived in the area for >40 yr. Fifty three sampling sites belonged to VC1, 75 to VC2, 86 to VC3, and 62 to VC4, corresponding to the approximate proportional areas covered by each vegetation class in each SL.

To characterize local soil conditions, three 10 cm-deep soil samples (ca. 400 g each) were taken at the centre, northern and southern limits of each sampling plot and subsequently pooled to obtain a single compound sample. The following physical-chemical properties were analyzed: pH in water (1:2 ratio), electric conductivity (EC; 1:5 extracts), cation exchange capacity (CEC; Olsen), soil organic matter (SOM, combustion or Walkley-Black), total nitrogen (N, Kjeldahl), available phosphorous (P, Bray), interchangeable potassium (K; with ammonium acetate), and soil texture (percent lime, clay and sand content, Bouyoucos) [39]. Finally, we recorded elevation and slope for each plot using a Digital Elevation Model (DEM, spatial resolution: 50 m) from the Instituto Nacional de Estadística, Geografía e Informática (INEGI).

Plant species were identified in the field by expert regional parataxonomists. Plant specimens of those species that could not be identified in the field were collected and identified by experts from the herbarium at the Center of Scientific Research of Yucatan. We categorized species according to two functional attributes: growth form (shrubs, trees and lianas) and dispersal syndrome (authochory, anemochory and zoochory) based on information obtained from the herbarium at the Center of Scientific Research of Yucatan (http://www.cicy.mx/ofer-tec-herbario/herbario), descriptions provided in the Flora of Veracruz (http://www1.inecol.edu.mx/floraver/inicio.htm), the Flora Mesoamericana (http://www.tropicos.org/Project/FM), the Flora of Barro Colorado Island (http://biogeodb.stri.si.edu/bioinformatics/croat/home), the Flora of Guatemala (http://archive.org/search.php?query=flora%20of%20guatemala), The Missouri Botanical Garden (http://www.mobot.org/mobot/fm/), and based on the expert opinion of regional parataxonomists.

### Spatial data

We used a principal coordinate of neighbor matrices analysis (PCNM, [47]) using the R software with the ‘spacemakeR’ library (version 2.10.1, R Development Core Team) to generate a set of explanatory spatial variables that represent the spatial structure of sampling sites. This set of variables (called PCNM vectors) represents a spectral decomposition of the spatial relationships among the sampling sites [48]. PCNM vectors are also uncorrelated variables that can be used as predictors in regression analysis to describe spatial relationships in community data, because they are not subject to multicollinearity [47]. This analysis was performed separately for each functional group. In each analysis we selected those PCNM vectors having positive, significant autocorrelation values (Moran's I; P>0.001) [48].

### Data analysis

We compared the relative magnitude of variation in woody species composition among dispersal syndromes and growth forms using Detrented Correspondence Analysis (DCA). This method indicates the variation in species composition in standard deviation units, which are directly comparable among datasets [49]. To assess the differences in species composition of each dispersal syndrome and growth form among vegetation classes we used an analysis of similarity (ANOSIM) in PRIMER-E 6.1.12 [50].

We performed a variation partitioning analysis [51] to evaluate the relative contribution of the abiotic environment (elevation and soil properties), stand age, and spatial structure of sampling sites on β-diversity for each dispersal syndrome and growth form. The total variation of each assemblage was dissected into five components: (1) a pure abiotic environmental effect, (2) a pure effect of spatial structure of sampling sites, (3) a stand age effect, (4) the combined variation explained by all components, and (5) the unexplained variation. To this end, we transformed species abundances using Hellinger's transformation and conducted a redundancy analysis (RDA) [52] in CANOCO 4.0 [53], down-weighing the most abundant species to reduce their influence on the analysis [54]. We recorded the proportion of the variation (R^2^) explained by all significant (*P*<0.05) variables of each component (i.e. soil properties, stand age and PCNM vectors) using forward selection with unrestricted Monte-Carlo permutations.

Additionally, we evaluated differences in species richness (α-diversity) among functional groups and vegetation classes using a generalized linear model (GLM) with a Poisson error distribution and a logarithmic link [55], [56], in which dispersal syndrome and growth form were nested within vegetation class. The best explanatory model was selected using backward stepwise selection starting from a global model [55]. *Post hoc* comparisons among vegetation classes, growth forms and dispersal syndromes were performed using contrast tests. These analyses were carried out in R v. 2.13.0 [57].

## Results

### B diversity by functional group and vegetation class

A total of 22,245 individuals belonging to 200 species and 50 families were recorded in the 276 plots (Table S1). Trees were by far the dominant growth form, both in terms of species richness and abundance, with shrubs and lianas lagging far behind. Zoochory was the most specious (but least abundant) dispersal syndrome, followed by autochocory and anemochory. Lianas were predominantly anemochorous, whilst shrubs were predominantly zoochorous and trees were more evenly distributed among and dominated all dispersal syndromes (Table 1).

**Table 1:** Species richness and abundance of woody plants by growth form and dispersal syndrome.

Dispersal syndrome	Growth form						Total abundance	Total richness
	Trees		Shrubs		Lianas			
	Abundance	Richness	Abundance	Richness	Abundance	Richness		
Anemochory	6431	17	8	4	1143	30	7582	51
Autochory	7594	42	324	14	19	3	7937	59
Zoochory	6013	61	575	23	138	6	6726	90
Total	20038	120	907	41	1300	39	22245	200

Spatial variation in floristic composition (β-diversity) was highest for shrubs (DCA ordination axes represented 19.13 SD), lowest for trees (6.97 SD), and intermediate for lianas (12.83 SD). With respect to dispersal syndrome, β-diversity was only slightly higher for the zoochorous guild (9.44 SD), than for the autochorous and anemochorous guilds (7.59 and 7.55 SD, respectively). The analysis of similarity (ANOSIM) revealed that tree species composition was similar between the youngest vegetation classes VC1 and VC2 (*P* = 0.15), but differed among all other classes (Table 2). Shrubs differed in species composition among all vegetation classes (*P*<0.011), except between VC3 and VC4, which were within the same range of successional age (16–70 years; *P* = 0.12). In contrast, liana species composition did not differ among vegetation classes, except between VC1 and VC4 (*P* = 0.02). Dispersal syndromes generally differed significantly among vegetation classes, except between the two youngest ones (Table 2).

**Table 2:** Results of ANOSIM comparisons of community composition among vegetation classes by growth form and dispersal syndrome.

Type of comparison		Growth form						Dispersal syndrome					
		Trees		Shrubs		Lianas		Anemochory		Autochory		Zoochory	
		*r*	*P*	*r*	*P*	*r*	*P*	*r*	*P*	*r*	*P*	*r*	*P*
All vegetation classes		0.28	<.01	0.11	<.01	0.014	0.17	0.18	<.01	0.24	<.01	0.18	<.01
Pairwise comparision	1–2	0.021	0.15	0.071	<.01	0.003	0.51	0.004	0.39	0.028	0.1	0.053	0.03
	1–3	0.29	<.01	0.21	<.01	0.031	0.24	0.241	<.01	0.128	<.01	0.212	<.01
	1–4	0.71	<.01	0.37	<.01	0.078	0.02	0.488	<.01	0.65	<.01	0.514	<.01
	2–3	0.16	<.01	0.05	0.011	0.009	0.18	0.129	<.01	0.113	<.01	0.055	<.01
	2–4	0.43	<.01	0.122	<.01	0.001	0.44	0.299	<.01	0.423	<.01	0.22	<.01
	3–4	0.24	<.01	0.016	0.12	0.006	0.34	0.053	<.01	0.25	<.01	0.229	<.01

### Species richness by functional group across secondary succession

**Figure 2:**
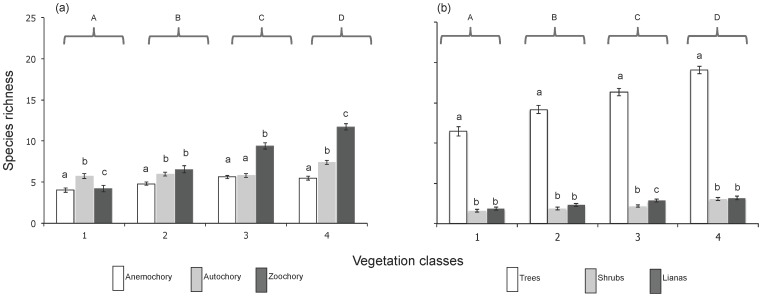
Differences in species richness by functional group among vegetation classes (upper-case letters) and among functional groups (lower-case letters). Different letters represent significant differences (*P*<0.001).

Species richness (α-diversity) differed significantly among vegetation classes, as well as among functional groups, and showed an increase with successional age (VC1<VC2<VC3) with a peak in VC4 (>15 yr-old forests on hills) (Fig. 2). Zoochory was the dominant dispersal syndrome in all vegetation classes, except for VC1 (3–8 yr-old forests), and followed the same general pattern shown by overall species richness (VC4>VC3>VC2>VC1). Autochory dominated in VC1, and showed higher richness than anemochory in all vegetation classes (*P*<0.001), except for VC3 (>15 yr-old forests on flat areas), where these syndromes were not significantly different. Trees were the dominant growth form, and showed the same general pattern as overall species richness (VC4>VC3>VC2>VC1). Shrubs and lianas had similar richness in all vegetation classes except for VC3, where there was a significant difference between them (*P*<0.001) (Fig. 2).

### Variation partitioning

Abiotic environmental variables (elevation and soil properties), successional age and spatial structure of sampling sites all influenced β-diversity, but accounted for less than one third of its total variation; furthermore, their relative influence differed among functional groups (Table 3). In terms of growth form, overall variation in species composition explained by all independent variables was greater for trees (29.2 % of total variation), compared to lianas (12.9%) and shrubs (10.3%). Moreover, the relative influence of explanatory variables varied considerably among growth forms. For trees, abiotic environmental variables accounted for most of the variation in composition that was explained by the model (51%), while shrubs were more strongly influenced by the spatial structure of sampling sites (46.8%), and lianas were similarly influenced by environmental variables and spatial structure (42.1% and 39.5%, respectively). Stand age accounted for around 12% of β-diversity of trees and shrubs, but had no significant influence on lianas. The single most important environmental variable for trees was soil organic matter (SOM; 74.5% of the variation due to abiotic environment), for shrubs it was cation exchange capacity (CEC; 76.5%) whereas for lianas it was pH (44.0%; Table 3).

**Table 3:** Variation partitioning (percentage) of β-diversity by plant growth form and dispersal syndrome through partial Redundancy analysis (RDA). Only significant variables (*P*<0.05) are included.

	Growth form			Dispersal syndrome		
*% Variation*	*Trees*	*Shrubs*	*Lianas*	*Anemochory*	*Autochory*	*Zoochory*
Total explained variation	29.2	10.3	12.9	27.4	31.2	20.4
Total unexplained variation	70.8	89.7	87.1	72.6	68.8	79.6
***Sets of variables***						
Environmental variables	51.0	36.2	42.1	48.8	59.0	47.6
Stand age	12.0	11.7		20.0	9.3	6.9
Spatial structure	27.7	46.8	39.5	24.2	22.4	40.2
Shared variation	9.3	5.3	18.5	7.0	9.3	5.4
***Environmental variables only***						
SOM^a^	74.5		18.0	61.9	82.1	81.4
pH	8.7		44.0	14.3	7.1	11.3
K	6.7	23.5		12.4	3.3	7.2
P					2.7	
% Sand content	6.0		24.0	11.4		
% Clay content	4.0					
CEC^b^		76.5			2.7	
Elevation			14.0		2.2	

a SOM: soil organic matter; ^b^CEC: cation exchange capacity.

Regarding dispersal syndromes, explanatory variables were slightly more strongly associated with the autochorous and anemochorous guilds (31.2% and 27.4%, respectively), than with the zoochorous guild (20.4%). Across dispersal syndromes, abiotic environmental variables (elevation and soil properties) consistently accounted for most of the variation in species composition (47.6–59%), followed by spatial structure of sampling sites (22.4–40.2%) –although, in the case of zoochory, the percentage of variation explained by abiotic variables and spatial structure was similar (47.6% and 40.2%, respectively). Forest stand age had a stronger influence on anemochorous species (20%), compared to autochorous and zoochorous ones (9.3% and 6.9%, respectively). Soil organic matter (SOM) was consistently the single most important environmental variable, accounting for 61.9–82.1% of the variation in composition due to abiotic environment across dispersal syndromes (Table 3).

## Discussion

We found marked differences in the magnitude and correlates of β-diversity among growth forms and smaller differences among dispersal syndromes. Our results suggest that both dispersal limitation and environmental filtering contribute to the assembly of woody plant species, but their relative influence varies among functional groups – especially growth forms.

### B-diversity by dispersal syndrome and growth form

In agreement with our first prediction (P1), variation in floristic composition was greatest for shrubs and lowest for trees. Other studies have shown a positive influence of plant height on dispersal distance [58], [59]. This is logical for wind-dispersed species, but could also hold for zoochorous species, possibly reflecting systematic variation in the animal species that forage at different canopy heights; as well as for autochorous species, since taller plants tend to have broader crowns [59]. Thus one may reasonably expect plant height to be directly related to dispersal distance and inversely related to β-diversity – although this does not explain why β-diversity was almost twice as high for lianas as for trees.

The magnitude of β-diversity differed only slightly among dispersal syndromes, possibly reflecting wide variation in seed dispersal distance within each dispersal syndrome [59]. The greater magnitude of β-diversity for zoochorous species than for autochorous and anemochorous species provides only partial support to our first prediction (P1) based on species dispersal distance potential. As expected, the lowest value of β-diversity corresponded to anemochory. However, autochory had a similarly low value of β-diversity, which contradicts our prediction. This latter result may be related to factors other than dispersal capacity, such as the ability to resprout after frequent human disturbance, such as extraction of wood and palm leaves for construction, and slash and burn agriculture, which has been practiced in our study area for over 2000 years [60]. Resprouting allows plants to survive after disturbance [61], [62] and uncouples species composition from seed availability [63], this could help explain why species with low dispersal capacity can dominate throughout the landscape and across succession [39], thereby reducing variation in species composition (i.e. β-diversity). Some examples of autochorous species that have a strong resprouting capacity and are among the most abundant species across successional ages and topographic positions are *Caesalpinia gaumeri* Greenm., *Bauhinia ungulata* L., *Croton reflexifolius* Kunth, and *Leucaena leucocephala* (Lam.) de Wit.

Contrary to our first prediction (P1), zoochory showed the highest value of β-diversity. This is likely the result of a highly variable dispersal range among zoochorous plant species depending on dispersers' motility, body size, diet and home range, as well as on fruit traits and the availability of alternate food sources [59]. Furthermore, it has been found that few animal-dispersed seeds are brought into recently abandoned agricultural areas by frugivores, even when forest fragments are nearby, probably because of fruit scarcity, higher predation risk or the unfamiliar habitat that an open area represents for frugivores [64], [65], [66]. Therefore, frequent disturbance, such as that produced by traditional shifting cultivation (as practiced in our study area) could reduce the dispersal of zoochorous species, thereby increasing this guild's β-diversity. Moreover, clumping of seed deposition was reported to be significantly higher for zoochorous species compared to anemochorous and autochorours species [66], which could also help explain the higher β-diversity of zoochorous plant species found in this study.

### α- and β-Diversity by functional group across secondary succession

As predicted (P2), overall species richness increased across functional groups along secondary forest succession (Fig. 2). Moreover, zoochory dominated in late successional ages and it also showed the highest α-diversity overall. This result agrees with our second prediction and with previous findings in other tropical dry forests in Brazil [67] and Ecuador [68], as well as in tropical wet forests in general [69], [70]. Zoochory also showed a noticeable increase in richness during secondary forest succession, as we initially predicted and as has been previously reported in tropical wet and dry forests [69], [71].

Contrary to our second prediction, however, anemochory had the lowest species richness along forest succession and showed a slight tendency to increase with successional age. Our study site is a relatively short-stature (8–12 m) semi-deciduous forest, which could help explain in part this result, since a considerable percentage of woody plants (25–50%) retain their leaves throughout the year. Both factors could have a negative effect on the efficiency of anemochorous seed dispersal, since the advantage of this dispersal syndrome in dry forests is associated with higher wind circulation during the dry season both through the upper canopy and lower vegetation layers [67]. The increase in anemochoruos species richness with successional age may result from the incorporation of new species of long-lived pioneers, such as *Albizia tomentosa* (Micheli) Standl., as well as shade-tolerant species such as *Gyrocarpus americanus* Jacq.

Regarding growth form, we also found only partial support for our second prediction. Trees were the most species-rich growth form not only in late successional ages (as predicted), but throughout succession (Fig. 2b). Tree species richness also increased with successional age, as expected and as previously reported in tropical forests [69], [71], [72]. Contrary to our prediction, however, shrubs did not dominate in the early phases of succession, possibly because some shrub species, such as *Bunchosia swartziana* Griseb., *Aphelandra scabra* (Vahl) Sm., and *Chiococca alba* (L.) Hitchc. are shade-tolerant. Overall relative richness of lianas (19.4%) was similar to that reported in other Neotropical forests ∼20% [73]. Absolute and relative liana species richness increased slightly from early to more advanced successional age classes, as also reported for the Mata Seca in Brazil [74]. The increase in liana richness with successional age may be related to the increasing availability of large trees that can provide structural support [75], [76], combined with the relatively high light availability that characterizes tropical dry forests in general [77], [78].

Species composition of trees was similar between the youngest vegetation classes (VC1 and VC2), although species richness differed. Since most of our VC3 and VC4 sites were >30 y-old, this finding may be attributed to the dominance of a group of pioneer species during the first 15–20 years of succession, which are subsequently replaced by shade-tolerant species, as has been reported in several studies both in wet and in dry tropical forests [24], [28], [37], [79]. All other pairwise comparisons among vegetation classes differed in tree species composition, including VC3 and VC4 (>15 yr-old forests on flat areas and on hills, respectively), which indicates that topographic position also contributes to β- diversity of trees, as well as to α- diversity (Fig. 2b).

In contrast, species composition of shrubs differed among all vegetation classes except for VC3 and VC4, indicating that β-diversity of plants with this growth form varies with forest succession, but not with topographic position. Liana species composition did not differ significantly among vegetation classes (except between VC1 and VC4), suggesting that their β-diversity is relatively unaffected by forest succession and topographic position. This may be the consequence of the dominance across vegetation classes of three species of lianas that represented more than 50% of their total abundance. Besides, lianas typically produce deep roots and a highly efficient water-transport system, and they generally require well-lit environments for their successful establishment [80], [81]. These characteristics could help explain why lianas seem to be able to establish, grow and persist equally well under different topographic positions and successional age classes, since they may be able to cope with water stress and may not be limited by low light availability.

### Variation partitioning

The proportion of the total variation in species composition accounted for by the explanatory variables, as well as the relative importance of explanatory variables, varied markedly among growth forms and slightly among dispersal syndromes (Table 3). These results clearly indicate that β-diversity along with the factors that influence it depend on key functional attributes, such as growth form and, to a lesser extent, dispersal syndrome. As predicted (P3), environmental filters explained the largest percentage of β-diversity for trees and the lowest for shrubs. In fact, abiotic environmental variables contributed most to β- diversity across growth forms and dispersal syndromes, except for shrubs. The dominant role of environmental factors suggests that environmental filtering has a strong influence on β-diversity, as has been reported in previous studies [2], [15], [16], [82], [83], thus providing support to the niche partitioning theory.

Shrubs were the only guild for which the spatial structure of sampling sites explained a larger percentage of variation in species composition than environmental filters, suggesting that dispersal limitation plays a greater role on shrub β-diversity than environmental filters. This may be partly attributed to their relatively short stature, which may impose critical limits on their dispersal capacity, resulting in a strong spatial autocorrelation in species composition. Using the same data set and (local) spatial grain of this study, [14] found that both environmental variables and the spatial structure of sampling sites had similar influences on overall β-diversity of woody species. Our results clearly show that this general pattern masks major differences in the relative importance of environmental filters vs. dispersal limitation among growth forms. However, it is also possible that the greater influence of spatial structure of sampling sites relative to environmental filters on shrubs is partly related to key environmental variables, such as light and soil moisture availability, that were not measured, that are spatially structured, and that may have a greater influence on shrubs compared to trees and lianas – given the former shorter stature and generally shallower root system [84]. Further studies are required to evaluate this alternative explanation.

Dispersal syndromes showed fairly consistent patterns of variation partitioning of β-diversity, with a clear predominance of environmental filters, except for zoochory (Table 3). Contrary to our prediction (P3), the autochorous guild showed a slightly stronger association with environmental variables compared to the other dispersal syndromes. This prediction was based on the assumption that dispersal distance is generally greater for anemochorous species compared to autochorous ones, and that greater dispersal ability should allow species to colonize sites with the appropriate environmental conditions. Alternatively, autochorous species may indeed have the most limited dispersal distances, and assuming that environmental conditions are spatially structured, it is logical that short-distance dispersing, spatially aggregated species should show higher association with spatially autocorrelated environmental factors. This would require that the spatial pattern of species composition of autochorous plants closely matches the spatial structure of sampled environmental conditions, resulting in a large percentage of variation explained by the joint effect of environmental factors and spatial structure of sampling sites (*i.e*. a spatially structured environment). Although the variation explained jointly by environment and spatial structure was indeed highest for the autochorous guild, it was still small relative to the variation explained by pure environmental or pure spatial components (Table 3). Moreover, if autochorous species were indeed strongly limited by dispersal limitation, one would expect that the spatial structure of sampling sites would explain a large percentage of variation in species composition of this syndrome. Yet, this syndrome showed the lowest percentage of β-diversity explained by spatial structure of sampling sites. As previously discussed, this unexpected result could be partly attributed to the dominance of tree species with strong resprouting capacity, displaying different dispersal syndromes, predominantly autochory. On the other hand, the fact that the largest percentage of variation in species composition explained by spatial structure of sampling sites corresponded to the zoochorous guild could be partly attributed to the greater clumping of seed deposition that has been reported for this syndrome compared to the autochorous and anemochorous syndromes [59].

The effect of forest succession on β-diversity varied among functional groups. Stand age explained almost 12% of the total variation in species composition of trees and shrubs, but was unrelated to liana β-diversity. This result may be due to the capacity of lianas to establish, grow and persist equally well under different topographic positions and successional age classes, as discussed previously. Similarly, stand age explained less than 10% of variation in species composition of zoochorous and autochorous species, and 20% of the anemochorous β-diversity. In this same study area, [14] found that stand age explained only 3% of the total variation in species composition of all woody species. These contrasting results clearly illustrate the importance of considering different functional groups when evaluating the ecological factors that influence β-diversity.

Soil variables explained over half of the variation in tree species composition. Previous findings in the same study area showed that hills have a thinner soil layer with high rock and nutrient contents, whereas flat areas have deeper soils with high clay content [39]. These characteristics have a major influence on woody species composition, which is dominated by trees [39]. The most important environmental variable explaining β-diversity differed among growth forms: soil organic matter (SOM) for trees, cation exchange capacity (CEC) for shrubs, and pH for lianas. This result suggests that growth forms are limited by different soil variables, which is in agreement with niche partitioning theory. SOM was consistently the single most important environmental factor influencing β-diversity of all dispersal syndromes and of trees (Table 2). This result may be due to the positive influence of SOM on soil fertility and on the capacity of soils to hold water and incorporate nutrients [20], [60], [85]. Our results agree with findings from many tropical forests worldwide [15], [16], [86], suggesting that soil properties can be regarded as major drivers of variation in plant species composition (β-diversity).

To conclude, our results clearly show that the magnitude of β-diversity varied markedly among growth forms, but much less among dispersal syndromes. They also suggest that environmental filtering is an important factor explaining β-diversity of different functional groups, except for shrubs, for which dispersal limitation appears to be more important, possibly due to their low stature that may limit their dispersal capacity. Additionally, stand age clearly influenced α-diversity across functional groups, but showed a relatively low influence on β-diversity, possibly due to the prevalence of tree species with strong resprouting capacity. Finally, our results clearly show that considering different functional groups may reveal important differences in β-diversity patterns and correlates that are not apparent when one considers overall woody plant diversity. Such differences have important implications for ecological theory, and can inform conservation, restoration and management strategies needed to stem the relentless deforestation and degradation of tropical dry forests.

## Supporting Information

Table S1 (XLSX)
